# Sex Differences in Mental Health-Related Work Incapacity Across Occupational Sectors During the COVID-19 Lockdown in Spain

**DOI:** 10.3390/healthcare13101137

**Published:** 2025-05-14

**Authors:** Eva María Gutiérrez Naharro, Amalia Sillero Sillero, José Antonio Ponce Blandón, José Fernández Sáez

**Affiliations:** 1 Nursing Department, Escola Universitària Gimbernat, Adscrita a Universitat Autònoma de Barcelona, 08174 Sant Cugat, Spain; eva.gutierrez@eug.es; 2 Nursing Department, Faculty of Nursing, Physiotherapy and Podiatry, University of Seville, 41009 Seville, Spain; 3 Facultad de Enfermería, Campus Terres de l’Ebre, Universitat Rovira i Virgili, 43500 Tarragona, Spain; jfernandez@idiapjgol.info; 4 Servei Atenció Primaria Terres de l’Ebre, Institut Català de la Salut, 43500 Tarragona, Spain; 5 Unitat de Suport a la Recerca Terres de l’Ebre, Fundació Institut Universitari per a la Recerca a l’Atenció Primària de Salut Jordi Gol i Gurina (IDIAPJGol), 43500 Tortosa, Spain

**Keywords:** mental health, COVID-19, temporary work incapacity, occupational sector, sex

## Abstract

**Background**: The COVID-19 pandemic has exacerbated mental health challenges across occupational sectors, disproportionately affecting workers in essential and public-facing roles. Objectives: This study ai to identify the occupational sectors in Spain most affected by mental health-related Temporary Work Incapacity due to Common Contingencies during the first COVID-19 lookdown (14 March–21 June 2020) to examine sex-based differences and to analyze the associate economic burden. **Methods**: A descriptive, retrospective cross-sectional study was conducted using data from salaried workers affiliated with Asepeyo, a major Social Security mutual insurance provider in Spain. The sample comprised 5135 workers granted Temporary Work Incapacity due to mental health diagnoses during the lockdown period. Variables analyzed included sex, age, ICD-10 diagnosis, occupational sector, duration of medically certified leave, and estimated direct economic cost. A focused subsample of 2123 workers from the ten most affected sectors was also examined. **Results**: Generalized Anxiety Disorder was the most prevalent diagnosis (69.17%), followed by adjustment disorders and depressive episodes. Women accounted for 63.5% of the total sample and 80.6% of the most affected sectors, which included elderly care, retail, education, cleaning, and healthcare. The average cost per episode was EUR 2465.7, with longer leave durations observed in sectors characterized by high emotional and social exposure. **Conclusions**: Mental health-related disorders during the COVID-19 lockdown revealed marked sex-based disparities and sectoral vulnerabilities. Public-facing and care-related occupations experienced a disproportionate burden. These findings support the need to recognize certain mental health conditions as occupational diseases to develop targeted, gender-informed workplace mental health strategies.

## 1. Introduction

Since the World Health Organization (WHO) declared COVID-19 a pandemic in early 2020, individuals’ personal and professional lives underwent profound changes [1]. The pandemic significantly impacted workers’ mental health across various occupational sectors, particularly frontline healthcare professionals, who experienced extreme stress, burnout, and psychological distress [2]. Other essential workers, including those in the food industry, transportation, and warehousing, also reported heightened anxiety due to increased workloads and perceived infection risks [3].

In Spain, stringent lockdown measures were implemented to mitigate virus transmission, reshaping nearly every social and economic domain [4]. Many businesses either ceased operations or radically altered their work conditions, creating novel psychosocial risks that threatened employees’ well-being. Psychosocial risks, defined as job-related factors that may lead to psychological or physical harm [5], intensified as the pandemic disrupted established work structures [6]. These risks included extended working hours, increased job demands, inadequate organizational communication, role conflicts, and job insecurity. The National Institute for Occupational Safety and Health in Spain reported that the abrupt shift to telework introduced additional challenges, such as work–life imbalances, blurred personal–professional boundaries, and digital fatigue [7]. Simultaneously, on-site essential workers, such as those in healthcare, transportation, and retail, faced significant exposure risks, often with insufficient personal protective equipment (PPE) [8].

According to the WHO, work-related stress emerges when job demands exceed an individual’s coping capacity, resulting in harmful psychological or physiological responses [9]. During the pandemic, these stressors frequently overwhelmed traditional coping strategies, particularly among Spain’s frontline and essential workers who faced extreme occupational pressure, feared contagion, worried about infecting their families, had extended shifts, and carried the emotional burden of treating critically ill patients [10]. Other professional sectors were similarly affected: public transportation employees maintained close contact with commuters; logistics workers handled surging delivery demands, and office-based personnel adapted to an abrupt transition to remote work [11].

Job insecurity further exacerbated psychological distress, particularly for employees in hospitality, tourism, and other heavily restricted industries [12]. Temporary suspensions of employment reflected broader economic instability, often heightening anxiety, depression, and uncertainty about future job prospects [13]. This trend parallelled global reports, highlighting the pandemic’s amplification of pre-existing mental health disorders. The cumulative consequences ranged from mild anxiety and mood disturbances to chronic stress, burnout, and, in severe cases, suicidal ideation [12]. These adverse effects diminished productivity, increased organizational strain, and underscored the urgent need for workplace interventions to protect employees’ mental health [13]. Consequently, Spanish authorities prioritized the evaluation of emerging psychosocial hazards and the implementation of strategies to reinforce workers’ well-being [11], aligning with international recommendations from the International Labour Organization (ILO) [2] and WHO [14] for enhancing occupational health policies to address psychosocial risks.

The psychological toll of COVID-19 in Spain became particularly evident during the lockdown period. Mental health-related disorders increased by 13.46%, and the total duration of sick leave due to psychological conditions rose by 29.56% [15]. Notably, mental health conditions emerged as the second most frequent cause of prolonged ill leave (≥365 days), accounting for 15.73% of all cases—an increase of 28.20% compared to the previous year—leading to a 10.64% rise in incapacity extensions. In response, the Spanish National Institute of Social Security enacted extraordinary measures, such as expanding the scope of Temporary Work Incapacity (ITL) to include preventive isolation and mandatory confinement. It enhanced protection for vulnerable individuals [7]. However, pandemic-related stressors persisted, encompassing a fear of contagion, financial strain, and the psychological burden of prolonged uncertainty.

During the first COVID-19 pandemic, specific occupational sectors faced heightened psychological and occupational risks. Healthcare workers endured emotional strain, a risk of infection, extended shifts, and shortages of protective gear [2,10]. Education professionals adapted to remote teaching under uncertain conditions [7], while staff in retail, cleaning, and transportation maintained essential services under precarious conditions and with increased exposure [8]. These sectors, marked by intense interpersonal contact and emotional demands, were particularly vulnerable to mental health deterioration [2,3].

Recognizing these challenges is crucial for interpreting sector-specific patterns of mental health-related work incapacity during the crisis.

Temporary Work Incapacity due to Common Contingencies (ITCCs) refers to medically certified sick leave for non-occupational illnesses managed through the Spanish National Social Security system [15]. During the first COVID-19 pandemic, its scope was expanded to include preventive isolation and mandatory confinement, reflecting its critical role in workforce health protection [7,15].

According to the latest WHO data, the pandemic contributed to a 25% global rise in anxiety and depression [16]. Furthermore, evidence suggests that women experienced higher rates of anxiety and depression, both under ordinary circumstances and during the crisis. One possible explanation is the over-representation of women in severely affected economic sectors (e.g., service industries) and frontline healthcare roles (e.g., nursing) [17].

Consequently, many workplaces struggled to implement effective mental health strategies during the rapidly changing pandemic conditions. Fragmented or small-scale initiatives often proved insufficient to address systemic issues [2,3]. A multidisciplinary approach that combined psychology, clinical medicine, social sciences, and public health was necessary to fully understand the impact of occupational mental health to strengthen workplace resilience in the face of future crises [2,3].

In this regard, preliminary data suggested that women were disproportionately employed in sectors with high emotional demands and frequent public interaction, such as healthcare, education, cleaning, and retail [16,17]. These gendered occupational patterns likely increased their vulnerability to mental health deterioration during the pandemic. Consequently, conducting a sex-based analysis was essential to fully understand the occupational impact of COVID-19 on Temporary Work Incapacity due to Common Contingencies.

This study analyzed data from MCSS Asepeyo, a major mutual insurance company collaborating with the Social Security system and covering a broad and diverse workforce across Spain. Although broadly representative, it does not encompass the entire national working population [15].

The analysis focused on the ten occupational sectors most affected by mental health-related ITL cases during the lockdown period, enabling sector-specific and sex-based comparisons. Further methodological details are provided in the Materials and Methods Section.

Based on these considerations, we hypothesized that occupational sectors characterized by high emotional labour and interpersonal exposure would show a greater incidence of mental health-related ITCCs and that women would be disproportionately affected due to their sectoral distribution.

In light of these challenges, this study aimed to address the following research question: Which occupational sectors in Spain were most affected by mental health-related Temporary Work Incapacity during the first COVID-19 lockdown (14 March–21 June 2020)? What were the most prevalent mental health diagnoses; how did the impact vary by sex (male/female), and what was the associated economic cost by sector?

By identifying the sectors most affected and the primary mental health conditions involved and by analyzing sex-based differences and economic consequences, this research provides critical evidence to inform the development of more resilient occupational health policies and targeted mental health support strategies.

## 2. Materials and Methods

### 2.1. Design and Setting

This is a descriptive, retrospective, cross-sectional study conducted in Spain. The objective was to evaluate which occupational sectors were most affected by ITCCs due to mental health conditions during the first COVID-19 lockdown period from 14 March 2020 to 21 June 2020. The analysis focused on workers affiliated with the Social Security mutual insurance provider Asepeyo, identifying the most affected sectors and the most prevalent mental health diagnoses, with a breakdown by sex (male/female) and total associated economic costs by labour sector.

### 2.2. Study Population and Sample

The target population for this study included all workers affiliated with the MCSS Asepeyo in Spain during the national state of emergency declared due to COVID-19 (14 March to 21 June 2020). The sample consisted of patients who met the following inclusion criteria: (1) those who were on ITCC leave certified by the Public Health System with a mental health diagnosis and (2) whose medical leave was managed by MCSS Asepeyo during the national state of emergency. Patients were excluded if (1) they were already on ITCCs for psychiatric reasons before the first COVID-19 lockdown, (2) they had relevant mental health diagnoses but were not evaluated by the responsible healthcare personnel, or (3) based on clinical evaluation and review of medical records, it was determined that their psychiatric conditions were unrelated to pandemic-specific stressors (e.g., infection risk, confinement, occupational changes, or social isolation). To ensure sufficient statistical power for sector-specific and sex-based comparisons, we focused on workers from the ten occupational sectors with the highest number of mental health-related ITL cases. Approximately 60% of the original cases, corresponding to workers from less-represented sectors, were excluded from the final analysis. The final sample from the most affected sectors was 2123 workers. While this strategy allowed for a detailed and comparative sectoral analysis, it may limit the generalizability of the findings to sectors not included in the study.

The Spanish Public Health System and MCSS Asepeyo used a classification system that included only the primary mental health diagnosis, defined as the main condition leading to the issuance of the ITCC certification, in the analysis. Although comorbid psychiatric conditions were occasionally documented in clinical records, only the principal diagnosis was used in the prevalence calculations. This approach ensured internal consistency and prevented the duplication of cases, aligning with standard epidemiological practices. Further below, Figure 2 displays the flowchart representing the process used to select and analyze the top 10 occupational sectors most affected by mental health-related Temporary Work Incapacity (ITL) during the first COVID-19 lockdown.

### 2.3. Procedure and Data Collection

Data were extracted from the CHAMAN database, the official mutual insurance registry maintained by MCSS Asepeyo and authorized by the Spanish Social Security system. All data were collected under strict data protection protocols, complying with national data protection legislation (LOPD). Patient records were pseudonymized using medical record numbers, with diagnoses classified under the ICD-10 system. Personally identifiable data such as names, surnames, or identification numbers were inaccessible. The dataset included variables such as sex (male/female), age, diagnosis, occupational sector (classified according to CNAE codes), duration of medical leave, and total direct economic cost per sector. Diagnoses were grouped by mental health condition and analyzed according to occupational sector and sex. The total financial burden attributed to ITCCs in each sector was also calculated and incorporated into the analysis.

### 2.4. Occupational Sector Classification

The occupational sectors were classified according to the Spanish implementation of the CNAE-2009 (National Statistical Classification of Economic Activities), which is aligned with the European NACE Rev.2 (Statistical Classification of Economic Activities in the European Community). This standardized classification system ensures consistency and comparability across datasets and research contexts.

For this study, only the ten sectors with the highest number of ITCC cases due to mental health diagnoses during the COVID-19 lookdown were included in the analysis. These sectors encompass a broad range of activities, including wholesale and retail trade, elderly care, healthcare services, call centres, education, public administration, and building cleaning services. The description of the main occupational sectors affected is as follows:

° Wholesale/Retail Trade of Textiles, Clothing, Footwear, Perfumery, and Cosmetics: Customer-facing retail work in fashion and personal care industries, often involving public interaction, job insecurity, and unstable conditions.
° Assistance in Residential Facilities for the Elderly with/without Healthcare: Caregiving staff in nursing homes and long-term care centres under pressure from high exposure, mortality, and emotional strain.
° Other Retail Trade of New Items in Non-Specialized Establishments: General merchandise retail with continued in-person service during lockdowns, increasing exposure and stress.
° Call Centre Activities: Remote or in-office roles with high pressure, repetitive tasks, and workload intensification during the pandemic.
° Manufacturing and Wholesale/Retail of Pharmaceutical Products and Specialties: Involved in essential production and supply of medical products, facing rising demand and stress.
° General/Specialized/Hospital/Dental Medicine Activities: Frontline health professionals exposed to high emotional and physical burdens.
° Education (Pre-primary to University): Teachers adapting to remote learning, managing uncertainty, and providing emotional support.
° Public Administration and Legal Matters: Civil servants facing abrupt transitions in work dynamics and public responsibilities.
° Wholesale/Retail Trade of Meat, Dairy, Eggs, Fish, Seafood, Oils, Cereals, Seeds, Raw Tobacco, Beverages, and Semi-Finished Products: Essential supply chain workers operating under pressure and exposure risks.
° Building Cleaning Service: Key personnel in hygiene and infection control, often exposed to hazardous conditions and high workloads.

### 2.5. Statistical Analysis

Descriptive statistics were calculated for all variables. Frequencies and percentages were reported for categorical variables (e.g., diagnosis, occupational sector, and sex) and means with standard deviations for continuous variables (e.g., age and duration of ITCCs). Comparisons by sex were performed using the Z-test for proportions. The Mann–Whitney U test was used for nonparametric age and leave duration comparisons between the male and female groups. The total economic cost per occupational sector was also reported in euros. Statistical significance was established at *p* < 0.05. Analyses were conducted using SPSS version 26.0 (IBM Corp., Armonk, NY, USA).

### 2.6. Ethical Considerations

The study was approved by the MCSS Asepeyo Research Ethics Committee (Approval Code: 2022/48-MLA-ASEPEYO; Approval Date: 31 May 2022). All procedures were conducted in accordance with the ethical principles outlined in the Declaration of Helsinki (October 2013, Fortaleza, Brazil). Patient confidentiality and data protection were rigorously ensured, and no personally identifiable information was collected or used.

## 3. Results

### 3.1. General Overview of the Total MCSS Asepeyo Population

The sample included 5135 workers affiliated with MCSS Asepeyo who received Temporary Work Disability benefits for mental health reasons during the first COVID-19 lockdown in 2020. Women represented 63.5% (n = 3259) of the total sample, while men accounted for 36.5% (n = 1876). The mean age for the overall population was 44.0 years (SD = 10.7), with women showing a mean age of 43.4 years (SD = 10.6) and men showing a mean age of 45.1 years (SD = 10.9).

### 3.2. Prevalent Diagnoses of Mental Health

The most prevalent diagnoses identified in this study were 13, ranked from highest to lowest prevalence: Generalized Anxiety Disorder (F41.1) (69.17%), with 2252 women (69.10%) and 1300 men (69.30%); adjustment disorder with conduct disturbance (F43.2) (11.47%), with 351 women (10.77%) and 238 men (12.69%); and major depressive disorder, single mild episode (F32.0) (7.52%), with 236 women (7.24%) and 150 men (8.00%).

**Figure 1:**
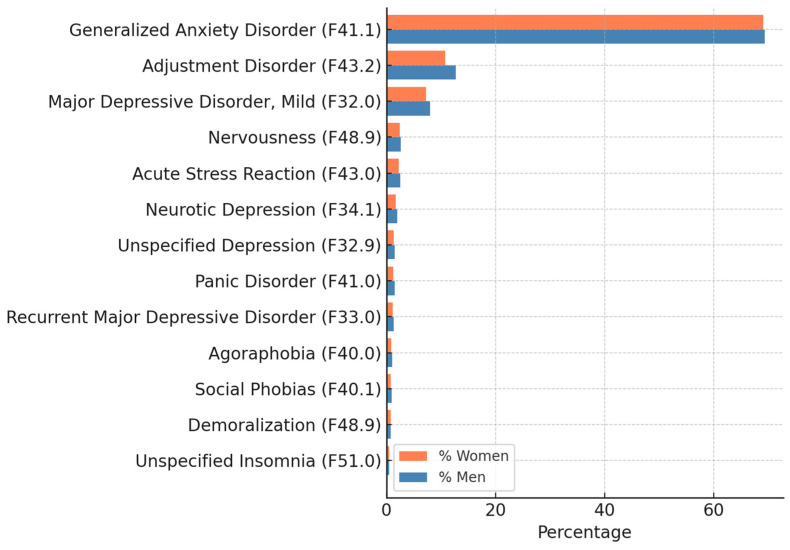
Prevalent mental health diagnoses by sex (ICD-10 codes) n = 5135.

The data presented in Figure 1 reflect the percentage distribution of each diagnosis stratified by sex, relative to the total number of certified cases within each group.

The analysis considered only the primary mental health diagnosis recorded in the ITCC certification, per the classification system used by the Spanish Public Health System. Although comorbidities (e.g., co-occurring anxiety and depressive disorders or eating disorders) were sometimes present in clinical documentation, only the principal diagnosis was used in the calculations to ensure consistency and avoid the duplication of cases (Figure 1).

Diagnoses included Generalized Anxiety Disorder (F41.1), adjustment disorder (F43.2), major depressive episode, mild (F32.0), nervousness (F48.9), acute stress reaction (F43.0), neurotic depression (F34.1), unspecified depression (F32.9), panic disorder (F41.0), recurrent major depressive disorder (F33.0), agoraphobia (F40.0), social phobia (F40.1), demoralization (F48.9), and unspecified insomnia (F51.0).

Note: Demoralization is not independently classified in ICD-10; it is associated with code R45.3 (other emotional disturbances).

### 3.3. Sociodemographic and Economic Profile of the Most Affected Sectors

**Figure 2:**
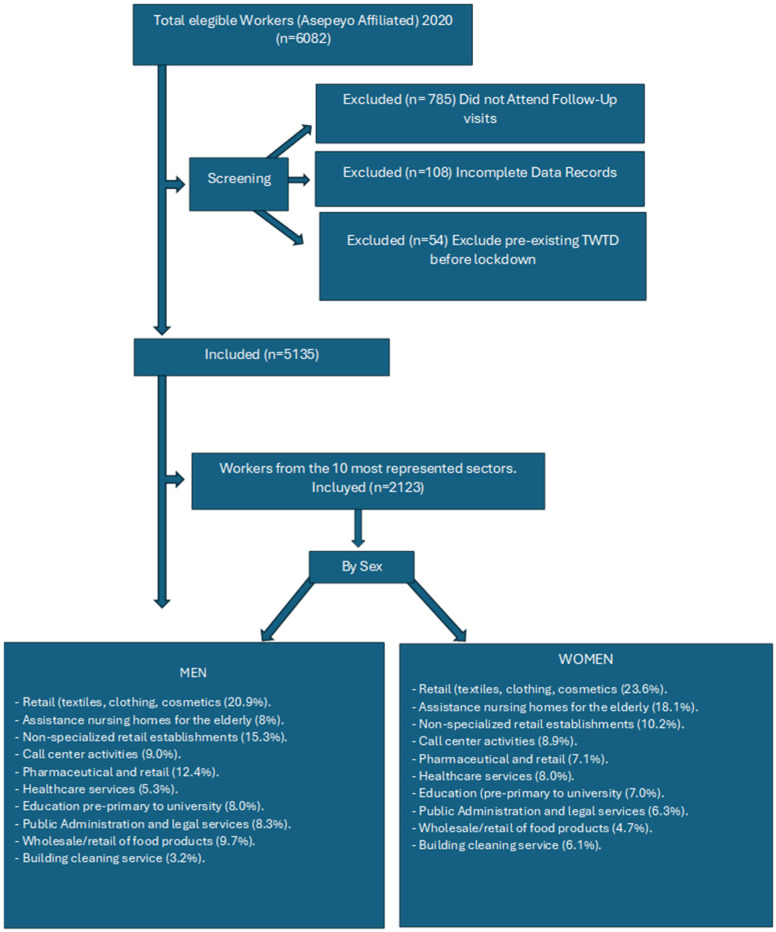
Flow chart of the data collection process.

This section focuses on the 2123 workers affiliated with MCSS Asepeyo, selected from the ten most affected occupational sectors during the first COVID-19 lockdown, who were granted mental health-related ITL (Figure 2 and Table 1).

The subsample is predominantly female (80.6%) and is largely concentrated in the general Social Security scheme (95.3%). The mean age was 43.9 years. Financially, the average daily regulatory base stood at EUR 55.0, and the estimated total cost per ITL case was EUR 2465.7. These figures reflect the economic and demographic profile of the most vulnerable occupational sectors during the pandemic.

### 3.4. Occupational Sector Distribution by Sex

**Figure 3:**
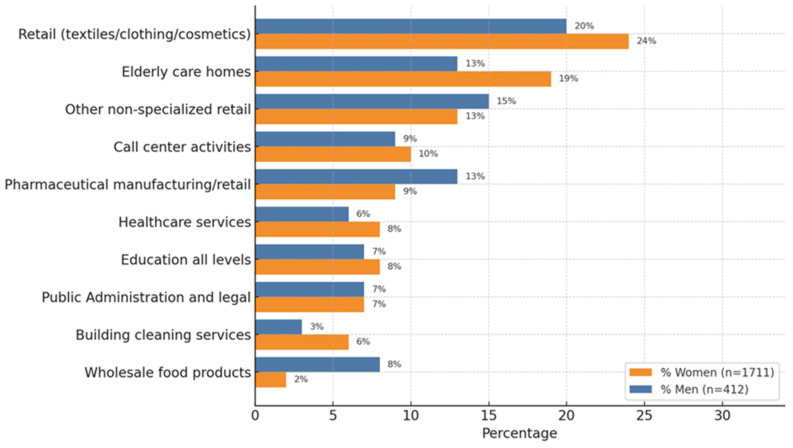
Distribution of workers by sector and sex (top 10 sectors, %); n = 2123.

The occupational sectors most affected by the pandemic in relation to mental health-related absences were those involving social interaction. Out of a total of 195 business sectors identified through the National Classification of Economic Activities (CNAE) codes, the 10 most affected sectors were included in this analysis. This chart shows the percentage distribution of women (n = 1711) and men (n = 412) across the top ten occupational sectors. The sectors are ordered by the percentage of women from lowest to highest. The data allow for a visual comparison between the two groups, highlighting gender-based patterns in occupational distribution (Figure 3).

The distribution of mental health-related ITL cases during the COVID-19 lockdown across the ten most represented occupational sectors was stratified by sex (men and women) and ordered in descending percentage.

Among men, the most represented sector was retail (textiles, clothing, and cosmetics) (20.9%), followed by other non-specialized retail (15.3%), pharmaceutical manufacturing/retail (12.4%), wholesale food products (9.7%), and call centre activities (9.0%). Other relevant sectors included public administration and legal services (8.3%), education (8.0%), elderly care homes (8.0%), healthcare services (5.3%), and building cleaning services (3.2%).

In contrast, among women, the leading sector was also Retail (23.6%), followed by elderly care homes (18.1%), other non-specialized retail (10.2%), and call centre activities (8.9%). Women showed a higher representation in building cleaning services (6.1%) compared to men, as well as substantial participation in healthcare services (8.0%), education (7.0%), and public administration and legal services (6.3%). Their representation in pharmaceutical manufacturing/retail (7.1%) and wholesale food products (4.7%) was lower than that of men.

This distribution highlights clear sex-based occupational patterns during the pandemic, with women more concentrated in caregiving, service, and retail roles, while men were more represented in sectors related to food distribution, pharmaceuticals, and administrative/legal services. These differences suggest varying occupational exposures and psychosocial stressors that may influence mental health outcomes by sex during public health crises.

### 3.5. Sex-Based Differences in Mental Health Diagnoses, Leave Duration, and Economic Impact

In addition to the occupational sector distribution, comparative analyses by sex were conducted regarding mental health diagnoses, duration of leave, and estimated economic cost. Comparative analyses between sexes were performed. Using the Mann–Whitney U test, age and ITL leave duration were compared between male and female workers. Statistically significant differences were observed, suggesting sex-based variations in the age distribution and work incapacity patterns.

Table 2 summarizes the key findings. Women were slightly more frequently diagnosed with Generalized Anxiety Disorder (F41.1) and adjustment disorders (F43.2), while men showed a marginally higher prevalence of major depressive disorder, single mild episode (F32.0). Significant differences were observed between sexes in age, the duration of leave, daily regulatory base, and estimated total cost per ITL episode. On average, women were younger, had longer leave durations, and generated slightly higher associated costs than men.

The comparative distribution of diagnoses (ICD-10), the mean duration of Temporary Work Disability (ITL), and the estimated economic cost per episode were stratified by sex.

## 4. Discussion

This study provides robust evidence of the significant impact of the COVID-19 pandemic on the mental health of workers in Spain during the lockdown period (14 March–21 June 2020). A total of 5135 cases of ITCCs due to mental health disorders were recorded, with a high concentration in occupational sectors characterized by public exposure, emotional labour, and precarious working conditions.

The ten most affected sectors—retail, elderly care, education, cleaning, healthcare and social services, call centres, supermarkets, transportation, hospitality, and public administration—accounted for a subsample of 2123 workers, of whom 1711 were women (80.6%) and 412 were men (19.4%). A marked disparity by sex was observed, with women being disproportionately represented in the most affected sectors, namely 90.4% of ITL cases in elderly care, 89% in cleaning, and 78% in education. Although these figures are biological in reporting, they must be interpreted in light of structural factors associated with gender.

The most affected sectors are historically feminized, linked to caregiving roles, and are typically characterized by lower wages, limited institutional recognition, and higher emotional demands [18,19]. These gendered labour patterns heighten workers’ vulnerability to psychological stress, particularly during public health crises, and are further compounded by the unequal distribution of unpaid domestic responsibilities [20,21].

The most prevalent diagnosis across all sectors was Generalized Anxiety Disorder (GAD), accounting for 69.17% of all ITL cases, followed by adjustment disorders and mild depressive episodes. This diagnostic trend aligns with international studies reporting increased psychological distress among workers during the pandemic, driven by fear of infection, social isolation, job insecurity, and intensified workloads in essential services [22,23].

The results observed support our initial hypothesis, which proposed that occupational sectors characterized by high emotional labour and interpersonal exposure would present a greater incidence of mental health-related ITCCs and that women would be disproportionately affected due to their sectoral distribution.

Our findings are consistent with the international literature. Recent studies confirmed the disproportionate mental health impact on workers in essential and public-facing sectors. For instance, a study on fashion retail workers in Spain reported elevated levels of burnout and mental workload among customer-facing employees [24]. Similarly, a European study focusing on self-employed workers has shown significant increases in anxiety and depression rates during the first COVID-19 lockdown, particularly in service-oriented professions [25]. A narrative review by Wang et al. [26] further emphasized the vulnerability of low-wage, public-facing workers to psychological disorders during the pandemic. This trend was not exclusive to Spain and reflected global structural inequalities across labour markets.

In addition, the results aligned with a longitudinal study [27] examining the economic and occupational impact of mental health-related ITL in Spain between 2020 and 2022, which confirmed the persistence of psychological absences beyond the initial lockdown phase and the emergence of new high-risk sectors [27]. Similar findings were reported by Navarro-Abal et al. [28], who documented a steady increase in mental health-related sickness absences across Spain, with a sharp rise during the first COVID-19 period.

The subgroup analysis of 2123 workers from the ten most affected sectors provided a more detailed demographic and economic profile of the most vulnerable employees: predominantly female (80.6%), with a mean age of 43.9 years, and an estimated average cost per ITL episode of EUR 2465.7. These data reflected not only the individual burden of mental health disorders but also their macroeconomic implications for the National Social Security system. Furthermore, sectors with a higher incidence of ITL also reported longer durations, especially for anxiety and depression, thereby exerting additional pressure on occupational health services [27].

Given these findings, there is a strong rationale for recognizing common stress-related mental health conditions, particularly GAD and adjustment disorders, as occupational diseases in sectors with elevated psychosocial risk. Such recognition would facilitate early intervention, reduce stigma, and improve access to compensation and rehabilitation mechanisms [18]. Workplace mental health strategies should also incorporate psychosocial risk assessments, emotional support systems, and structural improvements to working conditions, particularly in essential and high-demand occupations [19].

This study also underscores the importance of integrating a gender perspective into occupational health policies. While the data reflect disparities by sex, the overrepresentation of women in the most affected sectors is best explained by the gendered organization of labour shaped by cultural, social, and economic structures [20,21]. Effective interventions must, therefore, address not only clinical outcomes but also the broader systemic inequalities that increase workers’ exposure and vulnerability to psychological distress [29].

Although women represented 80.6% of the temporary mental health disability cases in this study, it is important to highlight that this higher proportion is likely linked to the occupational and social context of the pandemic rather than to an inherently greater psychological vulnerability. Many of the most affected sectors, such as elderly care, education, healthcare, and cleaning services, are historically feminized and involve high emotional demands and public-facing roles [20,21]. Furthermore, international studies have shown that during the first COVID-19 pandemic, women disproportionately assumed additional unpaid caregiving responsibilities, including childcare and eldercare, due to school closures and lockdown measures [22,23]. These combined occupational and domestic burdens likely contributed to greater psychological distress among women. Consequently, sex-based differences in work-related mental health outcomes should be interpreted in light of these structural inequalities rather than as biological differences in resilience [29]. Future research should explore the complex interactions between occupational roles, domestic responsibilities, and gender-related factors to better understand the differential mental health impacts observed during public health crises.

The discussion responds directly to this study’s objectives by identifying the most affected occupational sectors, the predominant mental health diagnoses, the sex-based distribution of impact, and the associated economic cost. These findings offer valuable insights for designing targeted, evidence-based occupational health policies and mental health support strategies in preparation for future public health emergencies.

### 4.1. Strengths and Limitations

A major strength of this study lies in its large and representative dataset. Moreover, the sector-specific and sex-disaggregated analysis provides a granular understanding of occupational risk patterns, offering valuable insights for public health and labour policies. This study contributes to the limited but growing body of empirical research on the intersection of sex, sectoral vulnerability, and mental health during global crises. It also complements national longitudinal studies covering subsequent phases of the pandemic. However, several limitations must be acknowledged. This study focuses exclusively on salaried workers affiliated with a single mutual insurance provider, which may limit the generalizability of the findings to self-employed individuals or workers covered by other institutions. Additionally, the cross-sectional design captures a specific time frame (March–June 2020) and does not account for the progression of psychological risk over time.

Moreover, the analysis focused on the ten occupational sectors with the highest number of ITL cases, which may limit the generalizability of the findings to sectors that were less represented in the sample. Diagnostic data are limited to medical records and do not include qualitative assessments of workplace conditions or individual coping mechanisms. Furthermore, classifying economic sectors may mask heterogeneity within broader categories, highlighting the need for future research to explore intra-sectoral differences. Despite these limitations, this study provides a robust foundation for understanding the impact of the COVID-19 pandemic on occupational mental health and identifying priority areas for targeted interventions.

### 4.2. Implications for Practice

The findings of this study highlight key areas for improving occupational mental health strategies. Conditions such as Generalized Anxiety Disorder and adjustment disorders should be considered occupational diseases in sectors with high psychosocial demands and frequent interpersonal contact, facilitating early diagnosis, compensation access, and destigmatization. Preventive measures should prioritize structurally vulnerable sectors characterized by job insecurity, emotional labour, and social exposure.

Occupational health services must have early detection protocols, psychosocial risk assessments, and accessible emotional support systems. Moreover, sex-based differences must be interpreted trough a gender-informed lens that addresses structural labour inequalities.

Finally, continuous longitudinal monitoring is necessary to identify persistent mental health effects, inform evidence-based policies, and strengthen the integration of mental health within occupational and public health preparedness frameworks.

In this regard, various international experiences offer valuable models. For example, Australia implemented sector-specific mental health interventions for frontline workers during the pandemic, including structured psychological support programmes and peer support networks [30]. Similarly, the United Kingdom expanded its “Mental Health at Work Commitment”, integrating mental health awareness training and early intervention protocols across multiple sectors [31]. These approaches emphasize the importance of effectively combining organizational, individual, and systemic strategies to address work-related mental health risks.

## 5. Conclusions

This study reveals distinct sex-based patterns in mental health-related work incapacity during the first COVID-19 lockdown in Spain. Women were significantly overrepresented in the ten most affected occupational sectors, reflecting their concentration in jobs with high emotional demands and social exposure. Generalized Anxiety Disorder emerged as the leading cause of psychological ITCC, contributing to both human and economic burdens. These findings underscore the need to strengthen occupational mental health policies, particularly in sectors with elevated psychosocial risk. Public health authorities and employers should implement preventive measures tailored to specific occupational environments and consider sex-based differences when designing interventions. Recognizing psychological disorders as occupational diseases in high-risk sectors could enhance early detection, reduce stigma, and improve access to appropriate support services.

Further research is warranted to explore the complex interplay between biological sex, gendered labour dynamics, and mental health outcomes using mixed-methods approaches that incorporate qualitative perspectives from affected workers. Additionally, developing sector-specific risk models could improve the prediction of psychological vulnerability based on occupational, emotional, and job security factors.

## Figures and Tables

**Table 1:** Sociodemographic and economic characteristics (n = 2123).

Variable	n (%)
Sex	
Female	1711 (80.6%)
Male	412 (19.4%)
Social Security Regime	
General Scheme	2023 (95.3%)
Self-Employed (RETA)	100 (4.7%)
	Mean (SD)
Age (years)	43.9 (10.7)
Daily Regulatory Base (€)	55.0 (27.9)
Estimated Total Cost (€)	2465.7 (1988.8)

**Table 2:** Sex-based comparison of diagnoses, duration of leave, and estimated cost (n = 2123).

Variable	Women (n = 1711)	Men (n = 412)	*p*-Value (Mann–Whitney U/Z-Test)
Generalized Anxiety Disorder (F41.1) (%)	69.10%	69.30%	0.920
Adjustment Disorder Single Mild Episode (F43.2) (%)	10.77%	12.69%	0.115
Major Depressive Disorder, Single Mild (F32.0) (%)	7.24%	8.00%	0.580
Mean Age (years)	43.7 ± 10.6	45.4 ± 10.9	0.003 *
Mean ITL Duration (days)	42.5 ± 25.3	40.1 ± 24.8	0.048 *
Mean Daily Regulatory Base (€)	54.3 ± 26.8	57.8 ± 29.1	0.010 *
Estimated Total Cost per ITL (€)	2520.4 ± 2000.3	2298.5 ± 1935.2	0.042 *

* *p* < 0.05.

## Data Availability

Aggregated data supporting this study’s findings are available upon reasonable request to the corresponding author, A.S.S., subject to review. These data are not publicly available due to privacy concerns and the potential for compromising research participant privacy/consent.

## Abbreviations

COVID-19	Coronavirus Disease 2019
ITL	Temporary Work Incapacity
CNAE	National Classification of Economic Activities
ICD-10	International Classification of Diseases, 10th Revision
ITCC	Temporary Work Incapacity due to Common Contingencies
ITL	Temporary Work Incapacity
WHO	World Health Organization
GAD	Generalized Anxiety Disorder
